# Immunomodulatory Impact of *Leishmania*-Induced Macrophage Exosomes: A Comparative Proteomic and Functional Analysis

**DOI:** 10.1371/journal.pntd.0002185

**Published:** 2013-05-02

**Authors:** Kasra Hassani, Martin Olivier

**Affiliations:** Departments of Microbiology & Immunology and Medicine, The Research Institute of the McGill University Health Centre, McGill University, Montréal, Québec, Canada; Institut Pasteur, France

## Abstract

Released by many eukaryotic cells, the exosomes are 40–100 nm vesicles shown to operate over the complex processes of cell-cell communication. Among the metazoan cell lineages known to generate exosomes is the mononuclear phagocyte lineage, a lineage that parasites such as *Leishmania* are known to subvert as host cells. We previously reported that mouse macrophage signaling and functions are modified once co-incubated with exoproteome of *Leishmania* promastigotes. Using mass spectrometry analysis, we were curious to further compare the content of purified exosomes released by the J774 mouse macrophage cell line exposed or not to either LPS or to stationary phase *Leishmania mexicana* promastigotes. Collectively, our analyses resulted in detection of 248 proteins, ∼50–80% of which were shared among the three sources studied. Using exponentially modified protein abundance index (emPAI) and network analyses, we found that the macrophage exosomes display unique signatures with respect to composition and abundance of many functional groups of proteins, such as plasma membrane-associated proteins, chaperones and metabolic enzymes. Moreover, for the first time, *L. mexicana* surface protease GP63 is shown to be present in exosomes released from J774 macrophages exposed to stationary phase promastigotes. We observed that macrophage exosomes are able to induce signaling molecules and transcription factors in naive macrophages. Finally, using qRT-PCR, we monitored modulation of expression of multiple immune-related genes within macrophages exposed to exosomes. We found all three groups of exosomes to induce expression of immune-related genes, the ones collected from macrophages exposed to *L. mexicana* sharing properties with exosomes collected from macrophage left unexposed to any agonist. Overall, our results allowed depicting that protein sorting into macrophage-derived exosomes depends upon the cell status and how such distinct protein sorting can in turn impact the functions of naive J774 cells.

## Author Summary

Secreted vesicles, such as exosomes, are now considered as an important route of communication among eukaryotic cells. Depending on the donor cell source and protein content, these vesicles are expected to distinctly impact the recipient cell properties. Here, three groups of exosomes released by the mouse macrophage cell line J774 exposed or not - naive exosomes - to either *Leishmania mexicana* promastigotes or to LPS were compared through proteomic analysis. Also, their biological activities on naive J774 macrophages were tested. Regardless of the source, the three groups of exosomes shared 50–80% of their proteins, although their relative abundances differed, especially those associated with the plasma membrane. Post exposure to one out of the three groups of exosomes, naive J774 recipient macrophages were compared for their profile of immune transcripts. Of note, whether they were exposed to either naive exosomes or to *L. mexicana*- induced exosomes, the naive J774 macrophages shared similar immune transcriptional signatures, the latter being distinct from the ones displayed by the macrophages exposed to LPS-induced exosomes. These data are discussed within the context of the unique cross talk that accounts for the early establishment of an immunomodulatory parasite such as *Leishmania* in its mammalian host.

## Introduction

Exosomes are 40–100 nm vesicles that are released by many eukaryotic cells. These vesicles are formed through invagination of the membrane into the multivesicular endosome (MVE) and can be released from the cell upon fusion of the MVE with the plasma membrane [1]. Although exosomes were once believed to be just packed with inert debris, current research suggests that along with other released vesicles, exosomes actually have an important part to play in different forms of long distance cell-cell communications [2].

Studies on exosomes derived from macrophages or dendritic cells (DCs) infected with bacteria shows that these exosomes are generally pro-inflammatory to naive macrophages, induce maturation of DCs and activate both CD4+ and CD8+ T cells [3], [4]. In addition, bacterial antigens such as glycopeptidolipids (GPLs) and immunogenic proteins have been found to be present on these exosomes and to be responsible for the pro-inflammatory nature of these exosomes [5], [6]. Therefore, exosomes introduce a novel class of communication among immune cells for antigen presentation and immune activation.

In contrast to bacterial pathogens, the biology of exosomes released from macrophages infected with immunomodulatory parasites such as *Leishmania* has not been previously studied. *Leishmania* parasites toggle between the extracellular motile and flagellated promastigotes, dwelling in the Phlebotomine sandfly and the roundshape nonmotile amastigotes residing in the phagolysosome of the mammalian macrophage [7]. These parasites have the ability to successfully parasitize macrophages thanks to their mechanisms for efficient inhibition of the signaling and microbicidal functions of their host. The hallmarks of these modulations are activation of protein tyrosine phosphatases (PTPs), inhibition of proinflammatory transcription factors NF-κB, AP-1 and STAT-1 as well as other critical signaling molecules such as JAK-2, IRAK-1 and MAP Kinases. Together, modulation of these molecules and pathways results in deactivation of macrophage microbicidal functions such as production of nitric oxide (NO) or proinflammatory cytokines such as TNF and IL-12. In addition to inhibition of macrophage functions, *Leishmania* infection renders the macrophage unresponsive to external stimulations such as LPS or IFN-γ (Reviewed in [8]). Moreover, we recently showed that GP63, the major surface protease of *Leishmania*, is able to gain access to the macrophage cytoplasm and directly cleave many intracellular targets, leading to inactivation of the macrophage [9]–[11]. Considering the modulatory nature of these parasites, studying the exosomes released from *Leishmania*-infected macrophages is of great interest. Importantly, it can shed light on how protein sorting to exosomes is altered following *Leishmania* infection and how it could affect targeting and functions of exosomes on other immune cells.

Different classes of proteins are now recurrently observed to be sorted into exosomes, such as proteins involved in adhesion (tetraspanins and integrins), vesicular trafficking (Alix, Tsg101), molecular chaperones (HSP 70, HSP 90), metabolic enzymes, and also cytoskeletal proteins [1]. Nevertheless, the content of exosomes is highly dependent on the cell type, its developmental status, as well as external stimulations [12], [13]. The combined function of those proteins on the recipient cell is still a subject of study. Still, exosomes have also been shown to carry molecules with known function in cell-cell interactions, such as MHC I or II, co-stimulatory molecules (e.g. CD80), and cytokines (e.g. TNF-α, TGF-β). The specific combination of surface molecules on exosomes could allow for specific targeting of the cytokines to distinct recipient cells. Additionally, infection with intracellular pathogens such as viruses or *Mycobacterium* species has shown to alter exosome content [3], [4], [14]. However, besides looking at specific markers or cytokines, alterations in the total proteome of macrophage exosomes after stimulation or infection have not been studied. Studying the proteome of exosomes is critical for understanding their biology, target selection and possible effects on recipient cells. Here we report the first comparative proteomic analysis of macrophage exosomes after LPS stimulation or infection with *Leishmania mexicana*. We show that the contents of macrophage exosomes go through dynamic changes following LPS stimulation or *Leishmania* infection. Furthermore, we show how these exosomes induce signaling and modulate expression of immune-related genes in naive macrophages.

## Materials and Methods

### Cell and parasite culture

J774A.1 murine macrophages were cultured in RPMI1640 medium (Wisent) supplemented with 10% heat-inactivated fetal bovine serum (FBS), streptomycin (100 µg/ml), penicillin (100 U/ml), and 2 mM L-glutamine at 37°C and 5% CO_2_. *L. mexicana* parasites were cultured in Schneider's Drosophila Medium (SDM) supplemented with 10% FBS at 25°C.

### Exosome collection and purification

Macrophages were stimulated with 100 ng/ml of LPS (Sigma), infected with stationary *L. mexicana* parasites at 1∶20 ratio or left untreated for 6 h. Macrophages were then washed with PBS and cultured for 24 h in RPMI medium supplemented with exosome-free FBS. Exosome-free FBS was acquired by overnight ultracentrifugation of FBS at 100,000 g for exosome collection. Culture supernatant was then collected and centrifugated at 4,000 g for 20 min to clear floating cells and debris. Supernatant was then passed through a 0.45 µm filter (Pall) to clear debris. Exosomes were pelleted by 1 h centrifugation at 100,000 g. Pelleted exosomes were resuspended, passed through a 0.22 µm filter (Pall) and washed in 20 mM HEPES pH 7.5. Washed exosomes were then resuspended in sterile HEPES 20 mM and kept at −80°C until use. In order to acquire ultrapure exosomes for mass spectrometry, following pelleting, resuspended exosomes were rapidly mixed with a cocktail of protease inhibitors (Roche) and washed in HEPES. Exosomes were then overlayed on a 0–2M gradient of sucrose and centrifugated for 12–16 h at 100,000 g. Fractions corresponding to 1.13–1.19M of sucrose were collected, passed through a 0.22 µm filter and pelleted at 100,000 g for 1 h. Exosomes were then resuspended in HEPES buffer and kept at −80°C until use. Purified exosomes were quantified using MicroBCA protein dosing assay (Thermo).

### Transmission electron microscopy

Exosomes were put on Fomvar Carbon grids (Mecalab, QC, Canada), fixed in 1% glutaraldehyde and stained with 1% uranyl acetate. Samples were visualized using FEI Technai-12 120 KV transmission electron microscope and AMT XR80C CCD Camera.

### Pseudomigration and protein digestion with trypsin

Proteins (5 µg) were loaded on an SDS-PAGE polyacrylamide gel containing 10% sucrose and run for 1 cm into the resolving gel. In-gel digestion was performed as described previously [15]. The gel lane was excised into 3 bands and each band was cut into 1 mm^3^ pieces. Gel pieces were first washed with water for 5 min and then dehydrated with acetonitrile (ACN). Proteins were reduced by adding the reduction buffer (10 mM DTT, 100 mM ammonium bicarbonate) for 30 min at 40°C, and then alkylated by adding the alkylation buffer (55 mM iodoacetamide, 100 mM ammonium bicarbonate) for 20 min at 40°C. Gel pieces were dehydrated and washed at 40°C by adding ACN for 5 min before discarding all the reagents. Gel pieces were dried for 5 min at 40°C and then re-hydrated at 4°C for 40 min with the trypsin solution (6 ng/µl of sequencing grade trypsin (Promega), 25 mM ammonium bicarbonate). The concentration of trypsin was kept low to reduce signal suppression effects and background originating from autolysis products when performing LC-MS/MS analysis. Protein digestion was performed at 58°C for 1 h and stopped with 15 µl of 1% formic acid/2% ACN. Supernatant was transferred into a 96-well plate and peptides extraction was performed with two 30 min extraction steps at room temperature using the extraction buffer (1% formic acid/50% ACN). All peptide extracts were pooled into the 96-well plate and then completely dried in vacuum centrifuge. The plate was sealed and stored at −20°C until LC-MS/MS analysis.

### LC-MS/MS

Prior to LC-MS/MS, protein digests were re-solubilized under agitation for 15 min in 10 µl of 0.2% formic acid. Desalting/cleanup of the digests was performed by using C_18_ ZipTip pipette tips (Millipore, Billerica, MA). Eluates were dried down in vacuum centrifuge and then re-solubilized under agitation for 15 min in 10 µL of 2% ACN/1% formic acid. The LC column was a C18 reversed phase column packed with a high-pressure packing cell. A 75 µm i.d. Self-Pack PicoFrit fused silica capillary column (New Objective, Woburn, MA) of 15 cm long was packed with the C18 Jupiter 5 µm 300 Å reverse-phase material (Phenomenex, Torrance, CA). The column was installed on the Easy-nLC II system (Proxeon Biosystems, Odense, Denmark) and coupled to the LTQ Orbitrap Velos (ThermoFisher Scientific, Bremen, Germany) equipped with a Proxeon nanoelectrospray ion source. The buffers used for chromatography were 0.2% formic acid (buffer A) and 100% ACN/0.2% formic acid (buffer B). During the first 12 min, 5 µl of sample was loaded on column at a flow rate of 600 nl/min and, subsequently, the gradient went from 2–55% buffer B in 100 min at a flow rate of 250 nl/min followed by a rapid increase to 90% buffer B and then came back at 2% buffer B for 10 min at a flowrate of 600 nl/min. LC-MS/MS data acquisition was accomplished using a eleven scan event cycle comprised of a full scan MS for scan event 1 acquired in the Orbitrap. The mass resolution for MS was set to 60,000 (at m/z 400) and used to trigger the ten additional MS/MS events acquired in parallel in the linear ion trap for the top ten most intense ions. Mass over charge ratio range was from 380 to 2000 for MS scanning with a target value of 1,000,000 charges and from ∼1/3 of parent m/z ratio to 2000 for MS/MS scanning with a target value of 10,000 charges. The data dependent scan events used a maximum ion fill time of 100 ms and 1 microscan. Target ions already selected for MS/MS were dynamically excluded for 25 s. Nanospray and S-lens voltages were set to 0.9–1.8 kV and 50 V, respectively. Capillary temperature was set to 225°C. MS/MS conditions were: normalized collision energy, 35 V; activation q, 0.25; activation time, 10 ms.

### Protein identification

Protein database searching was performed with Mascot 2.2 (Matrix Science) against NCBI *Mus musculus* and *Leishmania* protein databases. The mass tolerances for precursor and fragment ions were set to 10 ppm and 0.6 Da, respectively. Trypsin was used as the enzyme allowing for up to 2 missed cleavages. Carbamidomethyl and oxidation of methionine were allowed as variable modifications.

### Bioinformatic analyses of the proteomic data

Duplicates of separately analyzed sets of MS/MS data were used for calculation of the Exponentially modified protein abundance index (emPAI) values using emPAICalc web server (http://empai.iab.keio.ac.jp/) [16]. Mascot output files were uploaded to emPAICalc server and hits with minimum 3 peptides and minimum score of 20 were chosen as true hits for further analyses. Gene Ontology (GO) annotations of identified proteins were extracted using STRAP software [17]. Protein-protein interaction networks of the identified proteins were created using STRING database with default parameters and visualized using Cytoscape software 2.8 [18], [19].

### *In vitro* stimulation and infection

J774 macrophages were left un-treated, stimulated with 3–5 µg/ml of exosomes, 100 ng/ml of LPS or infected with stationary *L. mexicana* parasites at the ratio of 1∶20. Following the mentioned time-courses, cells were washed with PBS (3 times for the infected cells) and then lysed with appropriate lysing reagent as described below.

### Western blotting

Following *in vitro* stimulation, cells were lysed in a Western blotting lysis buffer. Proteins were dosed by Bradford reagent (Biorad) and run on SDS-PAGE according to standard methods. Proteins were blotted to Hy-bond nylon members (Amersham) and were detected by antibodies against actin, tubulin, PGK1, PABP, ERK, phospho-ERK, JNK, phospho-JNK, p38 and phospho p38 (all from Cell Signaling), or anti-phosphotyrosine clone 4G10 (Millipore). Anti-mouse or anti-rabbit and anti-rat antibodies conjugated to horse-radish peroxidise (HRP) (Amersham) were used as secondary antibodies. Membranes were then visualized by ECL Western blotting detection system (Amersham).

### Electrophoretic Mobility Shift Assay (EMSA)

EMSA was performed as described previously [20]. Briefly, nuclear proteins were extracted using an isotonic and then a hypotonic buffer. Extracted nuclear proteins were incubated with radiolabelled consensus sequences of NF-κB (5′-AGTTGAGGGGACTTTCCCAGGC-3′), AP-1(5′-AGCTCGCGTGACTCAGCTG-3′) and SP-1 (5′-ATTCGATCGGGGCGGGGCGAGC-3′) (Santa Cruz, CA, USA) as non-specific control. Samples were run on a native 4% acrylamide gel. Following electrophoresis, gels were dried and autoradiography was performed using Kodak film.

### Densitometry

Densitometry was performed using ImageJ software (NIH). Mean grey values of bands (ratio of phospho-proteins against their respective total proteins in case of MAP Kinases) were acquired and then normalized against the non-treated sample.

### Quantitative real-time PCR (qRT-PCR)

J774 macrophages were left untreated, infected with stationary *L. mexicana* parasites at 1∶20 ratio, stimulated with 100 ng/ml of LPS or 5 µg/ml of exosomes for 8 h. Following stimulation, cells were washed with PBS, (3 times for infected cells) and were lysed in Tryzol reagent (Invitrogen) according to the manufacturer's instructions for RNA extraction. Extracted RNA was then cleaned-up using Qiagen clean-up columns. Clearance of possible genomic DNA contamination was performed using DNase I (Promega) according to manufacturer's protocol. 1 µg of total RNA was used for cDNA preparation using reverse transcriptase enzyme Superscript III (Invitrogen) and random oligo-hexamers (Invitrogen). Samples were then treated with *Escherichia coli* RNase H (Invitrogen) for clearance of RNA-DNA helices. qRT-PCR was performed using Qiagen SABioscience RT^2^ profiler arrays in a Strategene mx3000 thermocycler according to SABiosciences protocol. Results were analyzed by ΔΔCt method using the Qiagen qRT-PCR data analysis web interface.

## Results

### Purification of exosomes from LPS-stimulated and *Leishmania*-infected macrophages

To collect exosomes from *Leishmania*-infected macrophages, we infected J774 macrophages with stationary *L. mexicana* parasites for 6 h to confidently saturate all macrophages with parasites. We washed away non-internalized parasites by PBS and incubated the macrophages in exosome-free medium for 24 h (LEISHX). Similarly, we stimulated the macrophages with 100 ng/ml of LPS as a strong stimulant for 6 h, to compare its effect with the immunomodulatory properties of *Leishmania* (LPSX). As a negative control, we incubated non-treated macrophages in exosome-free media for 24 h (NILX).

**Figure 1:**
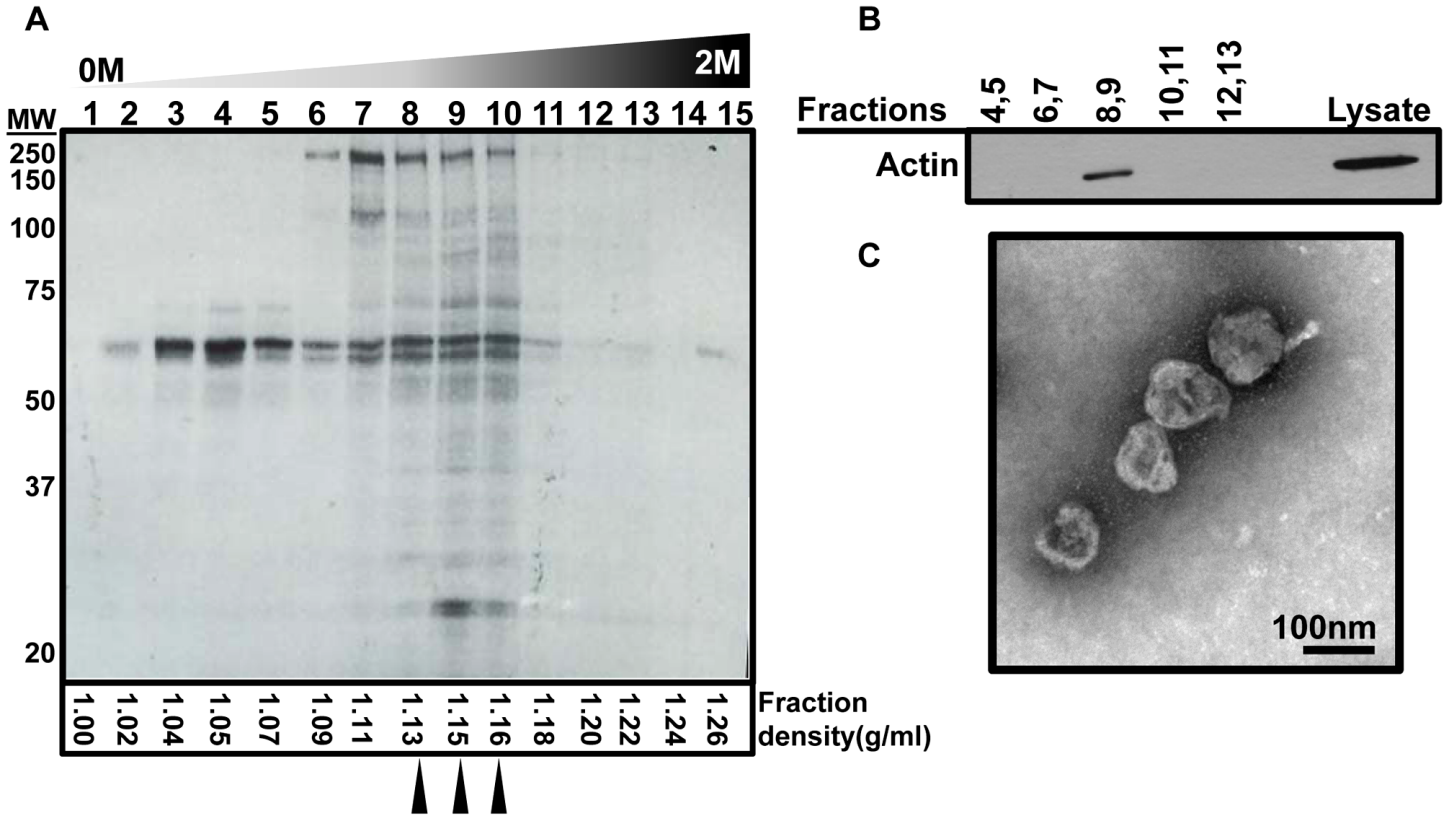
Purification of exosomes from culture supernatant of J774 macrophages. A. Silver staining of fractions resulted from sucrose density-gradient centrifugation of the crude exosome pellet. The majority of protein content is concentrated in fractions 8, 9 and 10 which correspond to proposed density of exosomes. Calculated density of each fraction (assuming linear sucrose distribution) is shown at the bottom. Arrowheads point to fractions that were picked for analyses. B. Western blotting on pellets acquired from the fractions in A show that actin, a protein known to be present in exosomes can only be observed in fractions 8 and 9. C. Transmission electron microscopy shows vesicles of about 100 nm in size confirming that the purified material is indeed exosomes.

Following incubations, we collected the conditioned medium and extracted the exosomes via multiple centrifugation and filtration processes as detailed in the materials and methods section. Exosomes settle at the density of 1.13 to 1.19 g/ml as can be seen in Figure 1A that shows a silver staining of fractions following sucrose density gradient centrifugation of exosomes. We further verified presence and purity of exosomes by western blotting against actin, known to be enriched in exosomes (Figure 1B). We did not observe any differences in density of exosomes after density gradient centrifugation of NILX, LPSX and LEISHX samples (data not shown). We recovered consistently but non-significantly less exosomes from LPS macrophages but observed no difference amongst LEISHX and NILX in terms of protein amount. Reduction in exosome release following LPS stimulation has been previously described to occur in DCs [21]. It is important to mention that our purification steps, especially involving filtration and density gradient centrifugation, would minimize contamination of our samples with other types of secreted vesicles. Nevertheless, transmission electron microscopy (TEM) of all samples showed only vesicles of 40–100 nm in size and morphology described for exosomes. We did not observe any vesicles of larger size suggesting that there was negligible if any contamination with larger secreted vesicles such as membrane vesicles [22]. No differences were observed among the samples suggesting that morphology and size of exosomes remain unaltered following LPS stimulation or *Leishmania* infection (representative TEM image shown in figure 1C).

### Comparative proteomic analysis of exosomes reveals major modulations following infection or LPS stimulation

**Figure 2:**
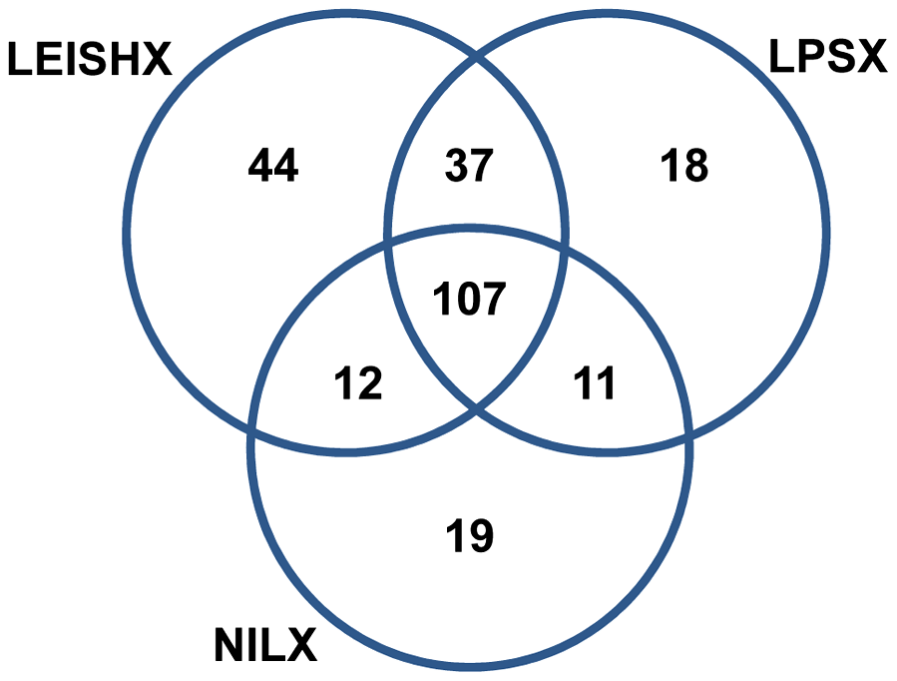
Common and unique proteins found in the three groups of exosomes by LC-MS/MS.

We performed mass spectrometry (LC-MS/MS) to analyze the content of the purified exosomes. Due to multiple washing steps in the purification, very few hits of contaminated serum proteins were found and were removed from the protein list. Detailed spectrum and peptide report as well as Pearson Coefficients comparing sample replicates against other samples are available in Supplemental File S1. Because peptide counts are not a reliable quantitative measure for sample comparison, we analyzed our proteomic data using the exponentially modified protein abundance index (emPAI) [23]. This method, calculates a ratio of observed to observable peptides, based on factors such as the conditions of the mass spectrometry analyses, protein biochemical properties and previously published empirical data. emPAI values are proposed to be linearly correlated to protein concentration and to give a more accurate estimate of protein abundance compared to simple peptide or spectral count [16], [23]. Only proteins with minimum 3 peptides and peptide score higher than 20 were considered as true hits. Also some proteins had to be removed because their relevant information was absent in the emPAI database. With these criteria, we ended up with a total of 248 proteins, which we used as the primary list for the rest of our analyses. The selected proteins and their calculated emPAI values are listed in supplemental files S1 and S2 respectively. We found 137 proteins in NILX, 173 proteins in LPSX and 200 proteins in LEISHX (Figure 2). We compared the list of the identified proteins against previously published exosome data at Exocarta database (www.exocarta.org, [24]) and found that the majority of the hits had been previously observed to be present in at least one group of exosomes (Supplemental File S1).

Since it had been previously reported that proteins from bacterial pathogens such as *Mycobacterium* can enter the macrophage exosomes, we also looked for *Leishmania* proteins in our MS/MS data. Interestingly, we observed positive hits for *Leishmania* surface metalloprotease GP63 in LEISHX exosomes and as expected not in NILX or LPSX (Supplemental File S3). To our knowledge, this is the first report of a protein from a eukaryotic parasite entering macrophage exosomes.

Interestingly, we observed that a high percentage of discovered proteins are shared among the 3 samples. 78% of proteins found in NILX, 62% of those found in LPSX and only 53% of proteins found in LEISHX were common among the 3 samples (107 proteins). While LEISHX had the highest number of unique proteins (44, 22%), LPSX and LEISHX had the highest percentage of shared proteins in between pairs (37, ∼20%). Therefore, we decided to compare the levels of abundance of the shared proteins among the samples.

**Figure 3:**
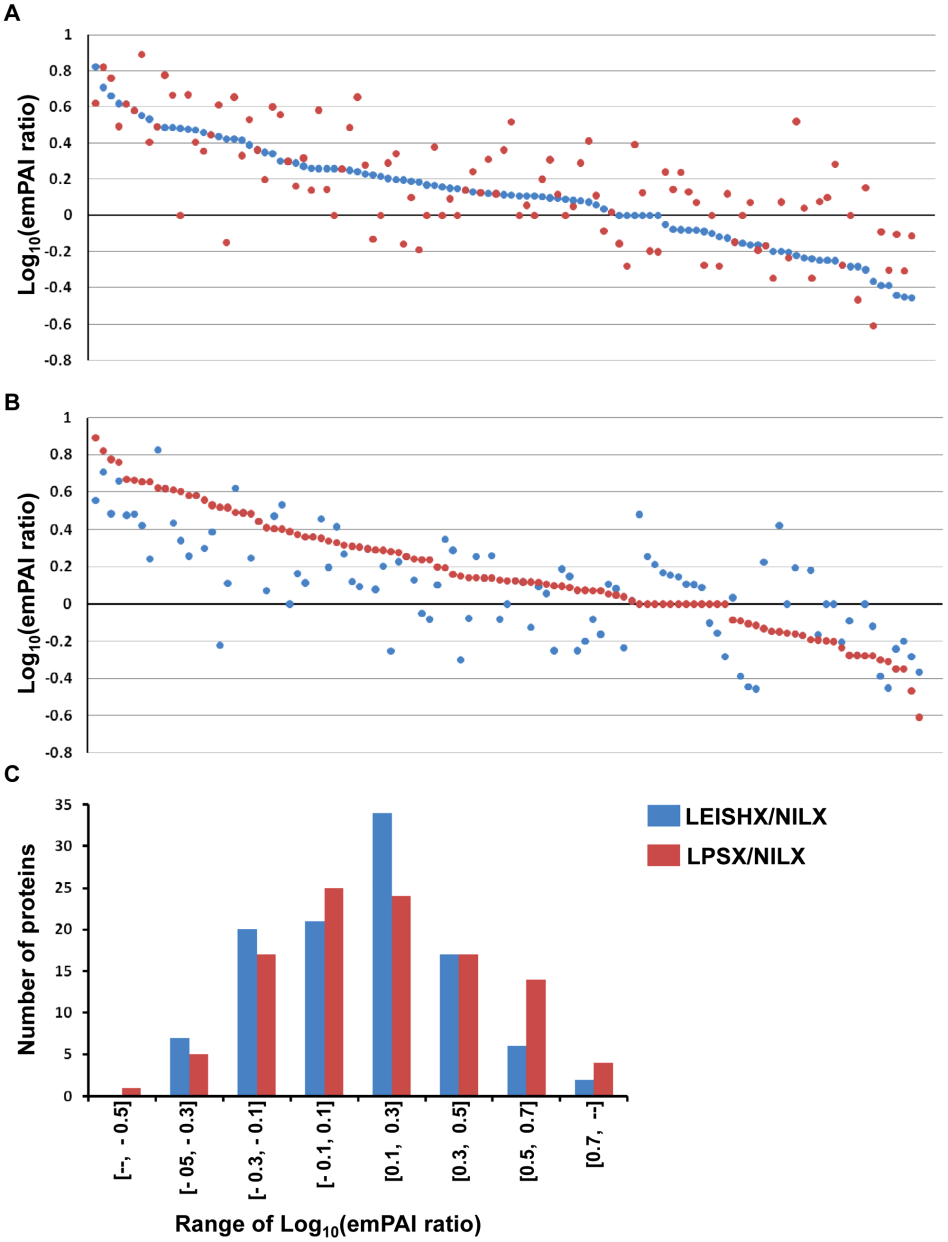
Comparison and distribution of emPAI ratios among NILX, LPSX and LEISHX exosomes. For each given protein, emPAI values in NILX, LPSX and LEISHX were calculated. LEISHX/NILX (red dots) and LPSX/NILX (blue dots) ratios were plotted in Log_10_ to show the general pattern of protein up or down regulation following *Leishmania* infection or LPS stimulation. The ratios are sorted from highest to lowest for LEISHX/NILX and LPSX/NILX in A and B respectively. C shows the frequency distribution of different ranges of LEISHX/NILX and LPSX/NILX emPAI ratios. Although a similar trend in patterns of modulation of protein content can be observed, many proteins have been modulated in opposite directions following LPS stimulation or *Leishmania* infection.

We compared the levels of abundance of the common 107 proteins by calculating their LEISHX/NILX and LPSX/NILX emPAI ratios (Supplemental File S2). Log_10_ of these ratios are plotted in Figure 3A and B, sorted from highest to lowest ratio for LEISHX/NILX and LPSX/NILX, respectively. The plots clearly show that although these proteins are shared among exosomes of naive, LPS-stimulated and *Leishmania*-infected exosomes, their abundance is greatly altered following these stimulations. In fact, very few proteins appear to have equal abundance, and a significant percentage have been altered more than 2 or 3 fold (Log_10_>0.3, Figure 3C). Interestingly, it appears that increase or decrease in abundance follows a similar trend in LPSX and LEISHX samples, whereby proteins increased in LEISHX are also increased in LPSX and vice-versa (Correlation coefficient = 0.72). This trend can also be observed when plotting the frequency distributions of LEISHX/NILX and LPSX/NILX emPAI ratios (Figure 3C). However, not all proteins follow the trend. In fact, ∼30% of proteins show opposite and divergent modulations between LPSX and LEISHX.

**Figure 4:**
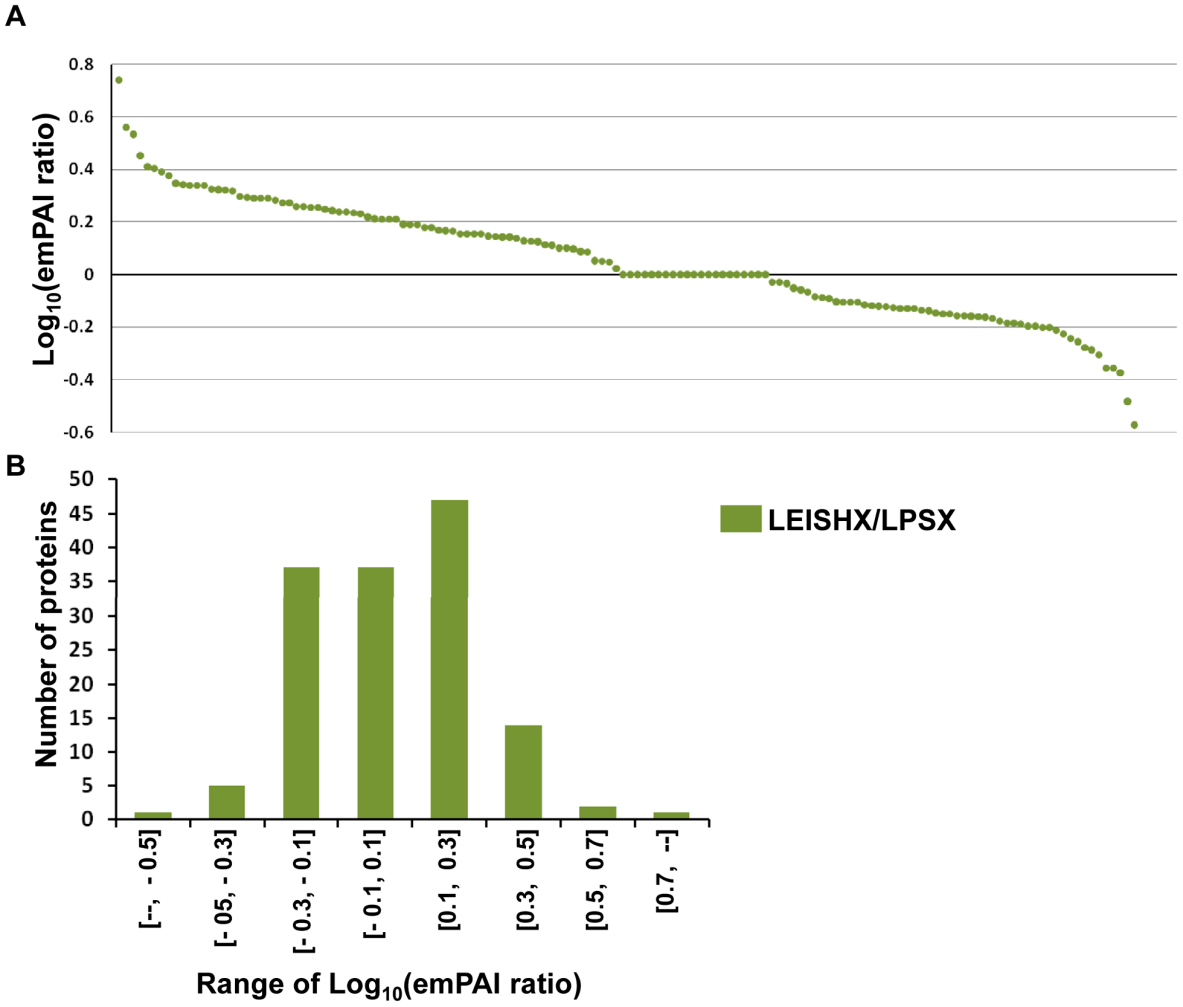
Distribution of LEISHX/LPSX emPAI ratios. A. The ratio of LEISHX/LPSX emPAI values. The ratios are sorted from highest to lowest for LEISHX/LPSX. B shows the frequency distribution of different ranges of the emPAI ratios.

We next compared modulation of proteins between LEISHX and LPSX themselves and also included the 37 proteins common between them to the 107 common proteins (Figure 4A). About half of the identified proteins have higher abundance in LEISHX, while about 35% have higher abundance in LPSX. However, frequency distribution of the emPAI ratios shows that the majority of the modulations lie within 2-fold difference (−0.3>Log_10_<0.3) (Figure 4B). Together these results show that *Leishmania* infection and LPS stimulation induce a similar trend of modulations in protein abundance in macrophage exosomes; although significant differences exist amongst the two types of stimulations.

### Gene Ontology and Network Analysis suggest modulation of specific protein groups

Various groups of proteins are sorted into exosomes. Having seen the modulations in protein abundance, as well as in the presence/absence of proteins among samples (Figures 2–4), we used Gene Ontology (GO) classification of proteins to look at the cellular localization, function and biological processes of groups that were up- or down-regulated. For simplicity, we merged the proteins unique to one sample with the proteins up-regulated in the same sample. We chose proteins with 1.5 fold or more difference in their emPAI value (−0.15>Log_10_<0.15) as the ones that are modulated between two samples. It is worthy to mention that since we chose proteins with minimum of 3 peptides as our starting criteria for inclusion into analyses, and we used emPAI analyses for correction of MS error, we are confident that 1.5 fold differences can have a real biological meaning. These cut-off lines resulted in labelling of 130 and 103 proteins as up-regulated in LEISHX and LPSX respectively. 51 and 60 proteins were also described as down-regulated in LEISHX and LPSX respectively. Finally, comparing LEISHX and LPSX resulted in 108 proteins higher in LEISHX compared to LPSX and 56 in the opposite. Between 20–25% of proteins in each comparison group were labelled as unchanged.

**Figure 5:**
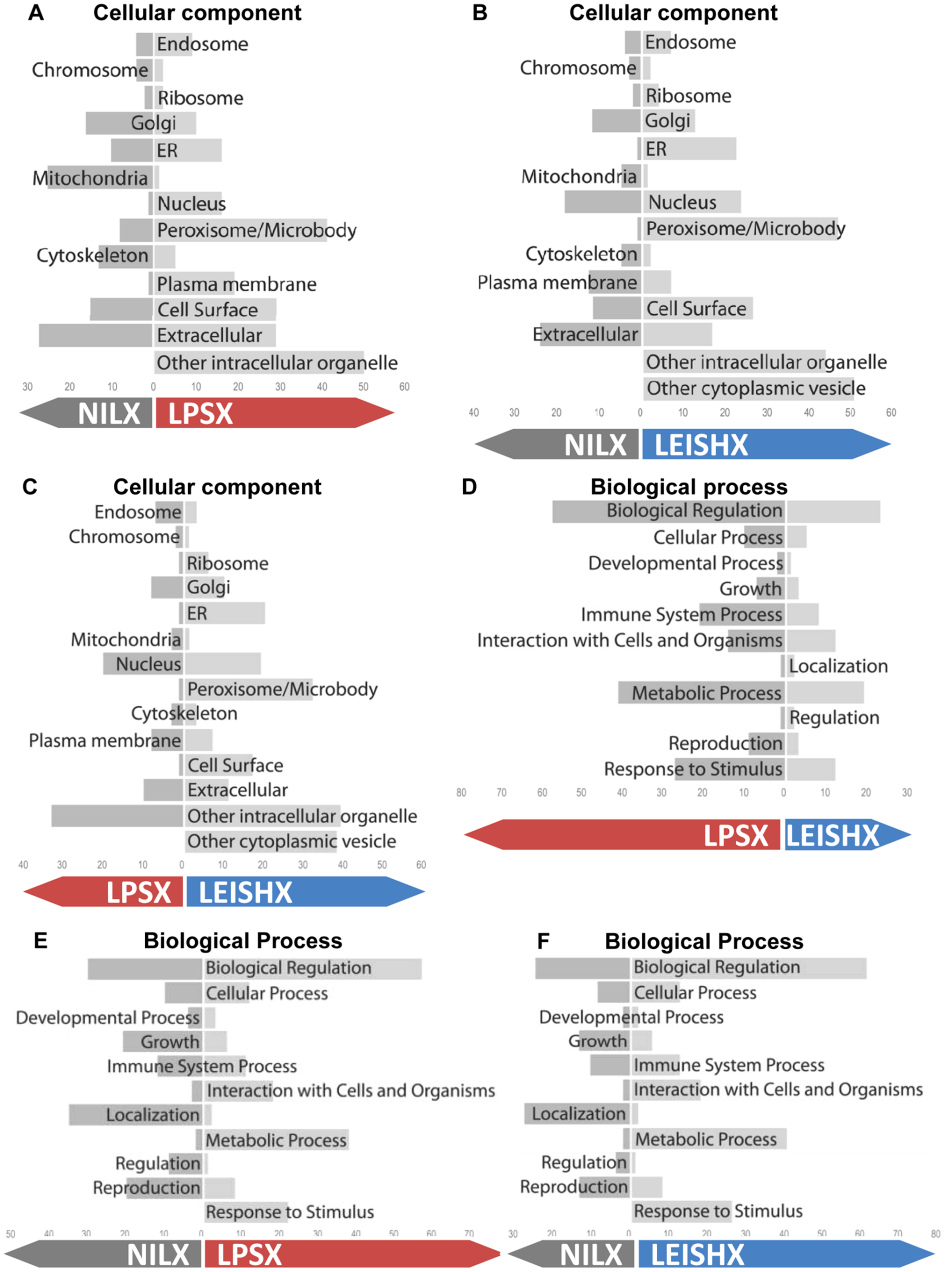
GO graphs of the proteins increased or decreased in NILX, LPSX and LEISHX exosomes. A, B and C compare the increase and decrease in cellular component associated GO terms among the three exosome samples. D, E and F show the changes with respect to biological processes. GO terms were acquired from Uniprot database via STRAP. Numbers on the bottom on each chart show number of terms associated with each group.

Comparative graphs of number of GO terms associated with each group of proteins show modulations in proteins associated with multiple functions, processes and cellular localizations in both LEISHX and LPSX (Figure 5). The comparative GO graphs of molecular function are presented in Supplemental File S4. Since more proteins are up-regulated than down-regulated in LEISHX and LPSX, it is not surprising that there appears to be generally more increases in GO terms associated with these samples. Although LPSX and LEISHX show similar patterns in up and down regulations compared to NILX (Figure 5A and B), direct comparison of LPSX and LEISHX reveals many differences in their associated GO terms. This shows that despite similarity, distinct functional groups and cellular processes are enriched in each set of exosomes.

**Figure 6:**
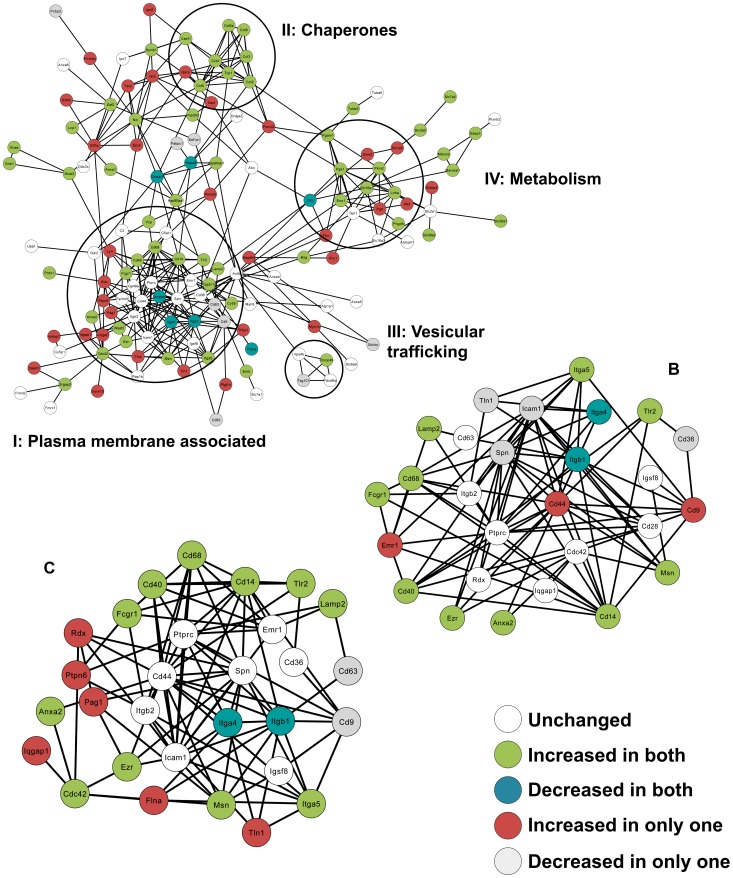
PPI networks of exosomal proteins. A. PPI network of proteins sorted in to LEISHX exosomes created via STRING database using default parameters. Interaction groups of proteins in related functional groups can be observed. B and C show the PPI network of plasma membrane associated proteins in LPSX and LEISHX exosomes respectively. Common and unique alterations in protein abundance can be observed.

Finally, to assess at the functional differences among the three samples at the protein level, we created exosome protein-protein interaction (PPI) networks using the STRING database for PPIs. Figure 6A shows the PPI network of the proteins identified in LEISHX (PPI networks of NILX and LPSX can be seen in Supplemental File S5). Looking closely at the PPI network, different functional groups of proteins known to be enriched in exosomes can be observed as interaction groups. For instance, circle I includes proteins associated with the plasma membrane and cell binding such as Integrin-β1 and β2 (Itgb1, Itgb2), CD63 and ICAM1, circle II includes chaperones such as members of the T-complex proteins, circle III includes proteins important in vesicular trafficking such as TSG101 and Alix (Pcdc6ip) and circle IV includes metabolic enzymes such as enolase, phosphoglycerate kinase (PGK1), lactate dehydrogenase (Ldha) and hexokinase (HK3). Other proteins usually present in exosomes such as signaling proteins, annexins and proteins involved in translation can also be observed in the PPI network. It can also be seen that actin (actb) is one of the proteins that connects these interaction groups with each other.

Since proteins associated with the plasma membrane generated the largest interaction group, we decided to compare those proteins among NILX, LPSX and LEISHX samples. Figures 6B and C show the proteins in the PPI network bearing the Cellular Component GO term, *plasma membrane* in LPSX and LEISHX respectively. Interestingly, many modulations occur with proteins involved in cell-cell contact. Levels of surface receptors or co-receptors such as Fc-γ-receptor 1 (Fcgr1), TLR2, CD40 and CD14 seem to increase with both stimulations. Integrins seem to be modulated with Integrin-β1 and Integrin-α4 decreasing and Integrin-β5 increasing, while Integrin-β4 remains unchanged. On the other hand, CD9 and CD44, two proteins important in cell-cell interaction and usually seen in exosomes, only increased in LPSX and not in LEISHX, while LEISHX shows an increase in PTPN6 (SHP-1), a PTP that we have shown to be modulated and activated after *Leishmania* infection [9], [25]. Together, our PPI network analysis of exosomes allows us to closely monitor the alterations in exosome surface that could play a role in exosome targeting and effector functions on recipient cells.

### Verification of the proteomic data

**Figure 7:**
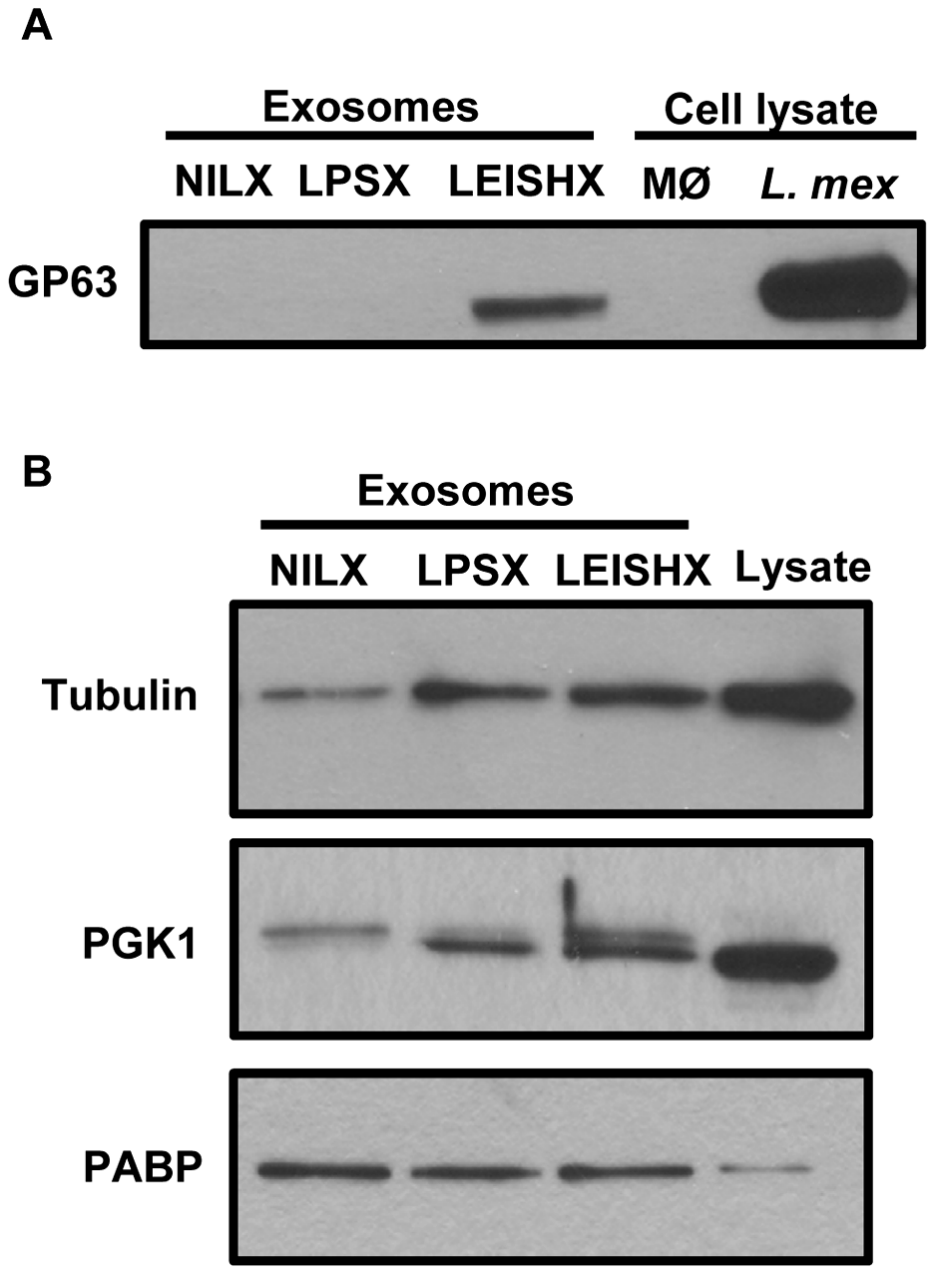
Verification of the proteomic data by Western blotting. A. *Leishmania* proteins B. Macrophage proteins. MØ: Macrophage.

We verified a number of the alterations in protein content in exosomes observed in our MS/MS results by western blotting. Firstly, we were able to confirm presence of GP63 in LEISHX (Figure 7A). Then, we looked at tubulin and PGK1, both of which that had shown higher emPAI values in both LPSX and LEISHX compared to NILX and we were able to observe their increase by western blotting as well (Figure 7B). In fact, we did not detect PGK1 in NILX by MS/MS that is probably due to its low abundance in these exosomes. Lastly, we looked at polyadenylated binding protein (PABP). Although emPAI values from MS/MS data showed reduction of in PABP in LPSX and LEISHX, we detected equal levels of this protein by western blotting. This reiterates the fact that results from MS/MS analyses should always be taken with caution.

### LPS and *Leishmania*-induced exosomes trigger activatory signaling in naive macrophages

**Figure 8:**
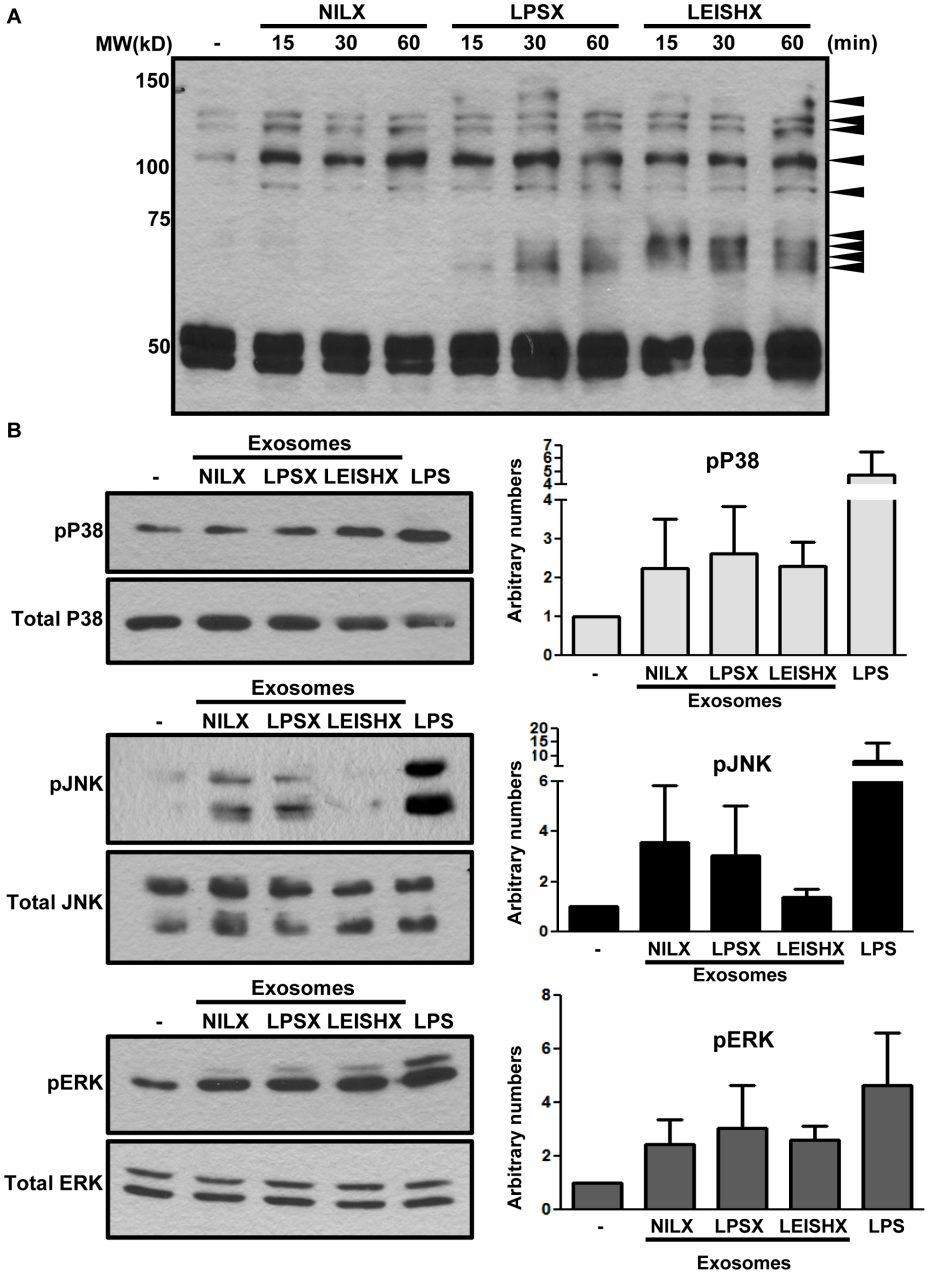
Macrophage exosomes induce Tyr phosphorylation and activation of MAP Kinases. A. 3–5 µg/ml of exosomes was given to naive J774 macrophages for the indicated time-points. Membranes were blotted with 4G10 anti-phosphotyrosine antibody. NILX, LPSX and LEISHX exosomes induce differential Tyr phosphorylation in naive macrophages maximizing at 1 h. Phosphorylations are stronger with LPSX and LEISHX compared to NILX. Arrowheads point to phosphorylations occurring following exosome stimulation. B. Naive macrophages were stimulated with exosomes or 100 ng/ml of LPS for 1 h. ERK and P38 MAP Kinases are phosphorylated after 1 h stimulation with NILX, LPSX and LEISHX exosomes. JNK is also phosphorylated after NILX and LPSX stimulation but not as strongly after LEISHX stimulation. The plots on the right side of each blot show average densitometric quantifications of 3 separate experiments, normalized against total protein and non-treated samples. Error bars show standard deviation.

Although the effect of exosomes on recipient cell function has been previously studied, the signaling pathways triggered leading to those functions have not been explored. To look at signaling pathways induced via exosome stimulation, we stimulated naive J774 macrophages with 3 µg/ml of pelleted and washed NILX, LPSX, LEISHX exosomes for 1 h and looked at patterns of general tyrosine phosphorylation, as well as phosphorylation of MAP Kinases ERK, JNK and P38. Figure 8A shows that stimulation with LPSX and LEISHX induces early Tyr phosphorylation of multiple proteins within as short as 15 min and increasing up to 1 h. Expectedly, stimulation with NILX does not induce a strong tyrosine phosphorylation compared to LPSX and LEISHX. Comparison of LPSX and LEISHX-stimulated cells identified both similar and unique Tyr phosphorylation patterns. We also looked at phosphorylation of MAP Kinases in naive macrophages within 1 h of stimulation with exosomes (Figure 8B). All 3 groups of exosomes appeared to induce phosphorylation of MAP Kinases, except for JNK which was not induced as strongly by LEISHX. Densitometric quantification of 3 separate experiments also supports weaker phosphorylation of JNK in response to LEISHX stimulation compared to other exosomes.

**Figure 9:**
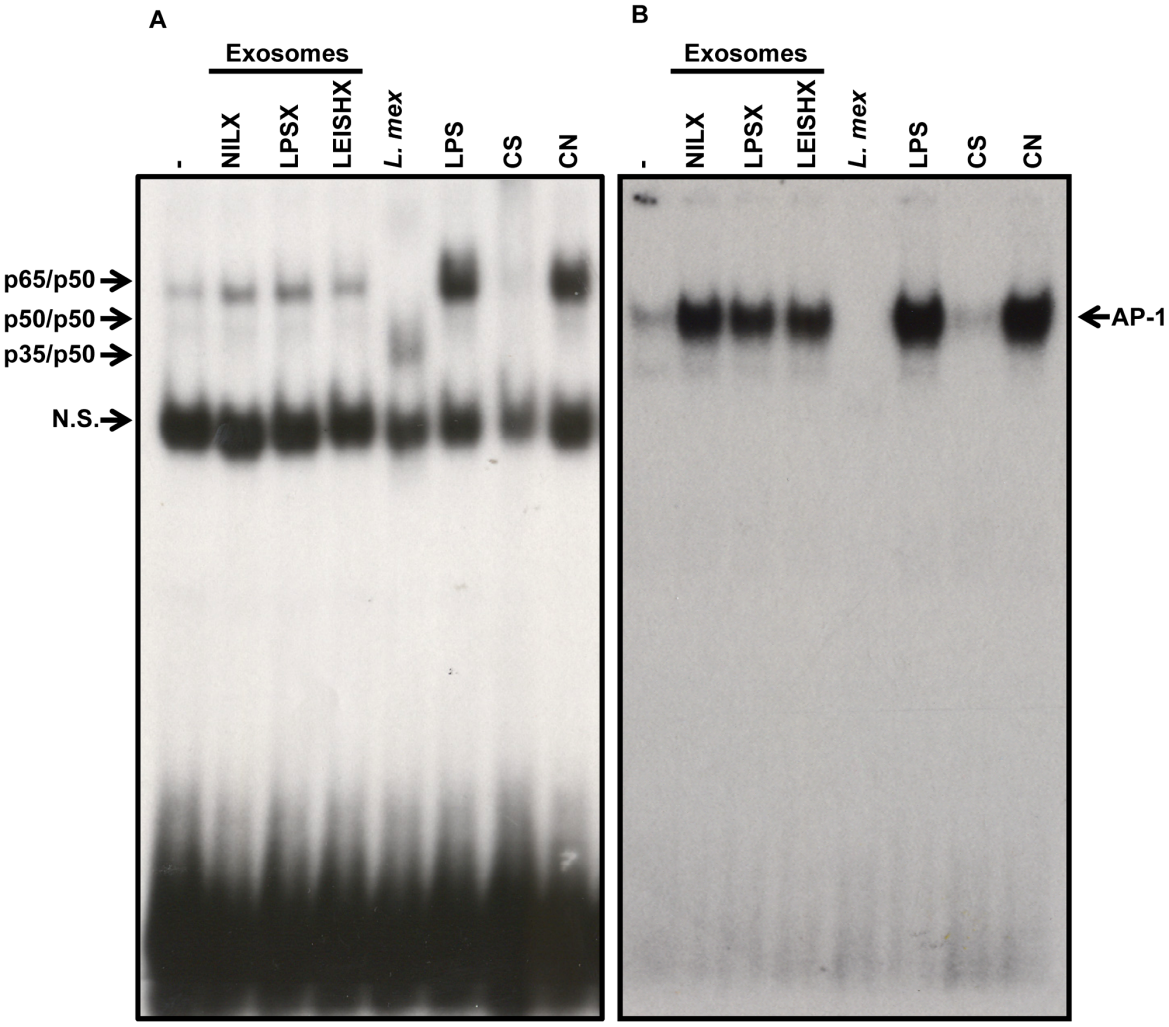
Exosomes induce translocation of NF-κB and AP-1 to the nucleus in naive macrophages. EMSAs show induction of nuclear translocation of NF-κB (A) and AP-1 (B) after 1 h stimulation with NILX, LPSX and LEISHX exosomes, although LEISHX exosomes appear to induce less activation of NF- κB compared to the other two. Infection with *L. mexicana* (*L. mex*) results in degradation AP-1 and cleavage of NF-κB. Stimulation with 100 ng/ml of LPS was used as a positive control. CS: Specific Control (100× cold oligo), CN: Nonspecific Control (100× cold consensus oligo for SP-1), N.S.: Non-specific band. Results are representative of 3 separate experiments.

Looking more downstream of protein phosphorylation, we studied activation of prominent pro-inflammatory transcription factors (TFs) NF-κB and AP-1 by exosomes. We stimulated naive macrophages with 3 µg/ml of exosomes for 1 h and performed EMSAs on extracted nuclear proteins (Figure 9). We observed that all 3 exosomes induce nuclear translocation of NF-κB and AP-1, although translocation of NF-κB is slightly less induced in response to LEISHX stimulation (Figure 9A). Infection with *L. mexicana* itself results in degradation of AP-1 and alteration of NF-κB as we had previously reported [20], [26]. We did not detect translocation of STAT-1 following exosome stimulation (data not shown). Overall, we saw that macrophage exosomes are capable of stimulating signaling molecules in naive macrophages, possibly resulting in distinct responses.

### Quantitative real-time PCR (qRT-PCR) reveals the downstream effects of exosome stimulation

**Figure 10:**
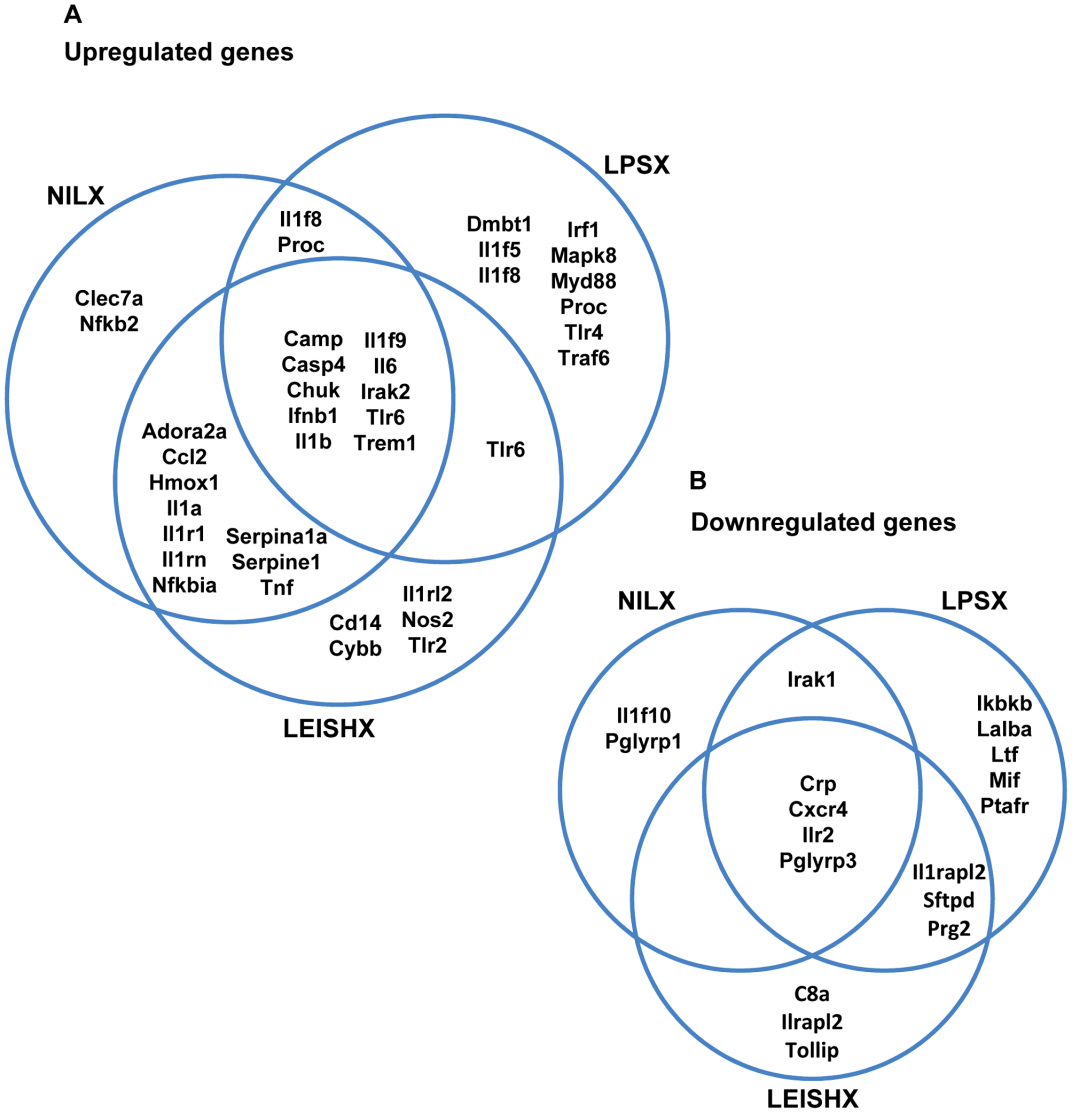
Exosome induced upregulation and downregulation of immune-related genes in naive macrophages measured by qRT-PCR. Macrophages were stimulated for 8 h with 5 µg of exosomes and modulation of gene expression was measured by qRT-PCR array. Venn diagrams show genes that were at least 2 fold upregulated (A) or downregulated (B) following stimulations. Results are average of duplicates.

The observed distinct activation patterns of signaling molecules and transcription factors by exosomes can lead to modulation of expression of many genes and different activation states in the recipient cell. To further scrutinize exosome-induced modulation of gene expression, we prepared cDNA from J774 macrophages stimulated with exosomes for 8 h and performed qRT-PCR using Qiagen SABiosciences RT^2^ Profiler arrays. Using these arrays, we measured modulation of expression of 90 immune related genes in exosome-stimulated macrophages. LPS (100 ng/ml) and *L. mexicana* infection were used as controls. The genes found to be at least 2-fold up-regulated or down-regulated after exosome stimulation are listed in Tables 1 and 2 (for complete data see Supplemental File S6). All 3 groups of exosomes appeared to be more stimulatory than inhibitory, as they induced more gene up-regulation than down-regulation. Especially, we observed up-regulation of pro-inflammatory cytokines such as IL-6 and IL-1 as well as certain interleukin receptors and TLRs. This is concurrent with our observations on activation of signaling molecules by exosomes. Figure 10 shows up-regulations and down-regulations that are shared following stimulation of macrophages with NILX, LPSX and LEISHX exosomes. The 10 genes that are induced by all 3 groups constitute ∼40–60% of the genes upregulated by each (Figure 10A). Interestingly, more than 80% of genes induced by NILX are induced by LEISHX as well, while this percentage for LPSX is only 50%. Additionally, LPSX induces the most unique set of genes compared to the other 2 groups. The same is true for the downregulated genes by LPSX (Figure 10B). This shows that NILX and LEISHX modulate gene expression more similarly compared to LPSX. Therefore, it suggests that exosomes from *Leishmania*-infected macrophages resemble more those of untreated macrophages than LPS-stimulated and activated macrophages. Infection with *L. mexicana* did not result in a stimulatory in terms of induction of immune-related genes and macrophage activation, in comparison to LPS and exosomes (Supplemental File S6). This is not surprising since *Leishmania* strongly modulates the pro-inflammatory transcription factors to avoid macrophage activation and establish its infection (Figure 9 and [10], [26]). Concurrently, the exosomes released from the infected macrophages also did not possess strong pro-inflammatory properties compared to naive exosomes.

**Table 1:** List of genes upregulated by at least one set of macrophage exosomes.

#	Name	Description	NILX	LPSX	LEISHX
**1**	Adora2a	Adenosine A2a receptor	**9.56**	−1	**23.86**
**2**	Camp	Cathelicidin antimicrobial peptide	**2.36**	**3.45**	**2.24**
**3**	Casp4	Caspase 4, apoptosis-related cysteine peptidase	**5.86**	**3.2**	**4.55**
**4**	Ccl2	Chemokine (C-C motif) ligand 2	**4.52**	1.36	**4.68**
**5**	Cd14	CD14 antigen	1.67	−1.11	**2.06**
**6**	Chuk	Conserved helix-loop-helix ubiquitous kinase	**2.7**	**2.38**	**3.63**
**7**	Clec7a	C-type lectin domain family 7, member a	**14.95**	1.38	1.35
**8**	Cybb	Cytochrome b-245, beta polypeptide	1.77	1.61	**2.62**
**9**	Dmbt1	Deleted in malignant brain tumors 1	1.04	**2.82**	1.28
**10**	Hmox1	Heme oxygenase (decycling) 1	**3.24**	1.65	**4.02**
**11**	Ifnb1	Interferon beta 1, fibroblast	**10.94**	**17.01**	**7.95**
**12**	Il1a	Interleukin 1 alpha	**155.1**	1.79	**358.71**
**13**	Il1b	Interleukin 1 beta	**149.8**	**3.13**	**920.75**
**14**	Il1f5	Interleukin 1 family, member 5 (delta)	−1.04	**7.51**	1.59
**15**	Il1f8	Interleukin 1 family, member 8	**4.68**	**2.33**	1.02
**16**	Il1f9	Interleukin 1 family, member 9	**13.28**	**5.71**	**13.66**
**17**	Il1r1	Interleukin 1 receptor, type I	**3.74**	1.98	**4.2**
**18**	Il1rapl2	Interleukin 1 receptor accessory protein-like 2	**4.19**	**2.24**	1.37
**19**	Il1rl2	Interleukin 1 receptor-like 2	1.23	1.62	**7.42**
**20**	Il1rn	Interleukin 1 receptor antagonist	**3.26**	−1.07	**4.47**
**21**	Il6	Interleukin 6	**7.82**	**7.33**	**48.06**
**22**	Irak2	Interleukin-1 receptor-associated kinase 2	**2.64**	**2.12**	**3.65**
**23**	Irf1	Interferon regulatory factor 1	1.41	**3.12**	1.21
**24**	Mapk8	Mitogen-activated protein kinase 8	1.11	**10.4**	1.44
**25**	Myd88	Myeloid differentiation response gene 88	−1.39	**3.94**	1.86
**26**	Nfkb2	NF-kappa B, p49/p100	**2.59**	−1	1.7
**27**	Nfkbia	NF-kappa B inhibitor, alpha	**2.96**	1.91	**4.02**
**28**	Nos2	Nitric oxide synthase 2, inducible	1.81	1.61	**6.95**
**29**	Proc	Protein C	**2.19**	**12.07**	1.06
**30**	Ptafr	Platelet-activating factor receptor	**2.98**	−1.15	**3.01**
**31**	Serpina1a	Serine peptidase inhibitor, clade A, member 1a	**2.04**	1.96	**2.02**
**32**	Serpine1	Serine peptidase inhibitor, clade E, member 1	**3.51**	−1.02	**3.56**
**33**	Tlr1	Toll-like receptor 1	1.36	**2.37**	**2.29**
**34**	Tlr2	Toll-like receptor 2	1.71	1.4	**2.53**
**35**	Tlr4	Toll-like receptor 4	−1.32	**3.03**	1.25
**36**	Tlr6	Toll-like receptor 6	**2.44**	**2.22**	**9.2**
**37**	Tnf	Tumor necrosis factor	**2.21**	1.61	**2.41**
**38**	Traf6	Tnf receptor-associated factor 6	1.45	**2.65**	1.55
**39**	Trem1	Triggering recepter of myeloid cells 1	**2.71**	**2.69**	**2.32**

Fold regulation against non-treated samples, calculated using ΔΔCt method, normalized against a panel of 3 house-keeping genes.

Results are average of 2 replicates. Numbers in bold are indicative of at least 2-fold upregulation.

Genes that are at least 2-fold up-regulated by one of the exosomes are listed.

**Table 2:** List of genes down-regulated by at least one set of macrophage exosomes.

#	Name	Description	NILX	LPSX	LEISHX
**1**	C8a	Complement component 8, alpha polypeptide	−1.3	1.62	**−3.12**
**2**	Cxcr4	Chemokine (C-X-C motif) receptor 4	**−7.96**	**−89.37**	**−9.98**
**3**	Ikbkb	Inhibitor of kappaB kinase beta	−1.02	**−92.84**	−1.02
**4**	Il1f10	Interleukin 1 family, member 10	**−2.36**	−1.35	−1.96
**5**	Il1r2	Interleukin 1 receptor, type II	**−20.8**	**−9.89**	**−10.33**
**6**	Irak1	Interleukin-1 receptor-associated kinase 1	**−2.15**	**−2.07**	−1.85
**7**	Lalba	Lactalbumin, alpha	**−2**	**−3.15**	−1.64
**8**	Ltf	Lactotransferrin	1.49	**−4.65**	−1.07
**9**	Mif	Macrophage migration inhibitory factor	−1.88	**−2.72**	−1.52
**10**	Pglyrp1	Peptidoglycan recognition protein 1	**−2.45**	1.52	−1.5
**11**	Pglyrp3	Peptidoglycan recognition protein 3	**−4.75**	**−2.83**	**−2.88**
**12**	Prg2	Proteoglycan 2, bone marrow	1.01	**−2.61**	**−3.68**
**13**	Sftpd	Surfactant associated protein D	1.43	**−3.38**	**−4.17**
**14**	Tollip	Toll interacting protein	1.17	1.18	**−2.28**

Fold regulation against non-treated samples, calculated using ΔΔCt method, normalized against a panel of 3 house-keeping genes.

Results are average of 2 replicates. Numbers in bold are indicative of at least 2-fold upregulation.

Genes that are at least 2-fold down-regulated by one of the exosomes are listed.

Overall, we compared the modulations that occur in macrophage exosome protein content, as well as their effector function on recipient cells, following *L. mexicana* infection and LPS stimulation. These data provide a better understanding of biology of exosomes and host-parasite interactions of *Leishmania*.

## Discussion

Studies on different functions of secreted vesicles, especially exosomes have now established them as yet another route for cell-cell communication, especially among immune cells [1]. Furthermore, it is now clear that sophisticated mechanisms are involved in exosome formation and exosomal protein sorting [27], [28]. Importantly, different extracellular stimulations or infectious agents such as viruses, intracellular bacteria or protozoa have been shown to alter exosome release from their host cells [14], [29]. However, modulations in the protein content of exosomes following these stimulations, especially infection, had not been previously studied. Here we report the first comparative proteomic analysis of naive macrophage exosomes against exosomes produced following stimulation with LPS or infection with *L. mexicana*. We interestingly observed that around 50–80% of proteins are shared among NILX, LPSX and LEISHX exosomes and this includes proteins that are usually reported to be sorted into exosomes, such as proteins involved in cell-cell communication, folding, vesicular trafficking and signaling. Combining the unique hits and also nuances in the levels of the shared proteins, we were able to look closely at the alterations in the sorting of functional groups of proteins into exosomes following these stimulations (See Figures 5 and 6).

We used emPAI values for our proteomic comparisons, which is a method regularly applied to assist in quantitative analysis of label-free mass spectrometric data [30]–[32]. This method allowed us to better quantify the differences among NILX, LPSX and LEISHX and observe many alterations in the levels of common proteins (Figures 3–6).

Choi *et al.* recently created the PPI network of proteins in exosomes derived from human colorectal cancer cells. They suggested that interacting proteins form complexes and functional modules in exosomes; and that PPIs can be involved in exosomal protein sorting [33]. We observed similar functional groups as those of Choi *et al.* and we were able to see how changes in the status of the macrophage can alter the composition and also abundance of these functional groups. Unfortunately, to date the underlying reasons for presence or absence of many of these proteins in exosomes are unclear. Therefore, our study can help connect the dots between the status of the macrophage, the contents of the released exosomes and their effector functions. For instance, using PPI networks of exosomes we saw that increases in chaperones occurred in both LPS and LEISHX, although increases in metabolic enzymes was more evident in LEISHX (Figure 6). Plasma membrane associated proteins showed most differences among the three sample groups, which can suggest that most alterations in exosome content could most importantly affect its targeting of recipient cells [34].

We were able to show that *Leishmania* GP63 is sorted into exosomes of infected macrophages via both MS/MS analysis and western blotting. GP63 is a key and highly abundant virulence factor of *Leishmania* promastigotes and our lab has previously shown that this enzyme is able to gain access to the macrophage cytoplasm and nucleus very early following *Leishmania* infection [9], [10] to alter many signaling molecules of the macrophage. It is therefore possible that presence of only GP63 and not other *Leishmania* proteins in the macrophage exosomes is due to its direct entrance through the cytosol and not through the communication of the phagolysosome with the MVE. Another possibility is that GP63 is a contamination from the parasites and not macrophage exosomes. We believe this to be very improbable because *firstly*, the unphagocytosed parasites were taken out by multiple washes before the exosomes-collection media was put on the macrophages; *secondly*, we did not detect any other proteins that are enriched in *Leishmania* exosomes or exoproteome; and *thirdly*, the collected exosomes were washed multiple times during the purification process to avoid contamination with any CM content.

Previous studies on exosomes released from infected cells with viruses and bacteria such as Herpes Simplex virus, Epstein-Barr virus and *Mycobacterium* sp. also showed that proteins from intracellular pathogens could be sorted into exosomes [6], [14]. In addition, it was proposed that the pro-inflammatory properties of exosomes released from bacterially infected macrophages are due to presence of these molecules and their triggering of pattern recognition receptors (PRRs) on the recipient cells [3]. However, we did not detect any *Leishmania* proteins other than GP63 to be sorted into exosomes by MS/MS or western blotting of common immunogenic *Leishmania* proteins such as LACK [35] (data not shown). GP63 is not naturally immunogenic, but is rather an immunomodulatory protein (see [8], [36] for reviews). Therefore, this could in part explain the absence of strong pro-inflammatory properties in LEISHX compared to NILX exosomes. The reasons for absence of other *Leishmania* proteins in macrophage exosomes could be the possible modulation of the interactions between the phagolysosome and the MVE.

We observed that the macrophage exosomes were able to induce phosphorylation of signaling proteins and translocation of activatory TFs into the nucleus. It was intriguing to observe a reduction in JNK phosphorylation following stimulation with LEISHX but not other exosomes. We speculate that this could be due to transfer of activated PTPs from the infected to the naive macrophage. As we have shown previously, *Leishmania* infection results in activation of a number of macrophage PTPs in a cleavage-dependent manner [9], [20]. In addition, our proteomic results show that exosomes contain PTPs, especially SHP-1 levels were shown to be increased in LEISHX (Figure 6C). Therefore, these transferred and active PTPs could possibly be involved in dephosphorylation of JNK. Similar mechanisms might also participate in reduction of NF-κB nuclear translocations.

To see how the activation of the signaling molecules is translated into function, we measured regulation of expression of immune-related genes by NILX, LPSX and LEISHX exosomes. Interestingly, all exosomes showed a relatively stimulatory behaviour and induced the expression of cytokines such as IL-6, members of the IL-1 family as well as certain receptors such as Interleukin receptors and TLRs (Table 1 and Figure 10). Exosome-induced production of cytokines such as IL-6, IL-1 and TNF by macrophages and monocytes has been reported elsewhere as well [37], [38]. Nevertheless, we did not detect secretion of TNF or NO by the exosome-stimulated macrophages (Data not shown). This shows that production and release of these critical immune modulators could be regulated at multiple levels. Exosomes might provide one of the necessary signals for this purpose. The relatively weak immuno-stimulatory properties of LPSX exosomes compared to macrophages infected with bacteria in other studies (reviewed in [4]) could be because we stimulated the macrophages with only LPS rather than living bacteria. Although it has been reported for bacterial antigens to be sorted into exosomes, this might not occur as much with LPS. Thus, LPSX exosomes might not be as immuno-stimulatory as exosomes derived from bacterially infected macrophages. Still, it was interesting to observe that the members of the LPS downstream signaling pathway, namely TLR4, MyD88 and TRAF6 to be up-regulated by LPSX, maybe priming macrophages for more sensitive detection of LPS in the environment.

The fact that we observed a pro-inflammatory behavior for NILX exosomes poses a question on its physiological relevance. However, one should bear in mind that there are fundamental shortcomings in the *in vitro* exosome function studies. First, there is limited knowledge on the physiological concentration and half-life of exosomes *in vivo*; thus most studies looking at exosomes *in vitro* usually have to use arbitrary concentrations. Additionally, the concerted effect of exosomes from various sources together with other factors such as cytokines and growth factors might lead to different outcomes from what observed *in vitro*.

Gene-expression regulation patterns of NILX and LEISHX looked alike (Figure 10A). This is in contrary to the exosomes released by bacterially infected macrophages, where they have been shown to have strong pro-inflammatory properties, compared to naive exosomes [3]–[5], [29]. However, *Leishmania*-induced exosomes do not show this behavior and appear to be more similar to naive exosomes, especially compared with LPS-induced exosomes. This is despite the fact that the macrophage is infected with an intracellular pathogen and its exosome content is altered. Therefore, *Leishmania* alters exosome production by the macrophage in a fashion that matches its other immune evasion virulence mechanisms.

One of the genes upregulated by NILX and very strongly by LEISHX is the Adenosine receptor 2a (Adora2a). Extracellular adenosine (usually generated during stress and inflammation via dephosphorylation of extracellular ATP) has been suggested to be a modulator of the innate immune response [39]. Especially, binding of adenosine to Adora2a in macrophages results in an inhibitory response consisting of increased IL-10 and reduced TNF release [40]. Interestingly, a recent study also showed that *Leishmania amazonensis* utilizes this receptor to antagonize inflammation and spread infection [41]. Thus, LEISHX upregulates Adora2a in bystander macrophages that could potentially be the next in line to be infected and more readily inhibited.

To conclude, we observed that LPS stimulation and *Leishmania* infection induce modulation of exosomal sorting into macrophages, especially with plasma membrane associated proteins. These modulations in turn altered effector functions and targeting of released exosomes. We were able to see that these modulations result in activation of signaling proteins and differential regulation of expression of immune-related genes. Our study also suggests that *Leishmania* could modulate the host's exosome machinery in its benefit; a virulence mechanism which needs to be further explored, especially by looking at antigen presentation by *Leishmania*-induced exosomes. Together our results give a better understanding of exosome biology in the innate immune system, connecting it to *Leishmania* host-parasite interactions.

## Supporting Information

File S1 **Spectrum and peptide information for the mass spectrometry data.**
(XLS)

File S2 **Calculated comparative emPAI values.**
(XLS)

File S3 **Spectrum and peptide information for the Mass spectrometry data, analyzed with the** ***Leishmania*** **database.**
(XLS)

File S4 **Other GO comparisons (Continued from** **Figure 5** **).** **A–C.** Pair-wise comparisons of GO terms associated with Molecular Function among NILX, LPSX and LEISHX are shown.
(PDF)

File S5 **High resolution PPI networks of NILX, LPSX and LEISHX exosomal proteins.**
(PDF)

File S6 **Complete qRT-PCR data.**
(XLS)
