# Supplementary material for: Immunomodulatory Impact of Leishmania-Induced Macrophage Exosomes: A Comparative Proteomic and Functional Analysis
Source: PLoS Negl Trop Dis. 2013 May 2;7(5):e2185. doi: 10.1371/journal.pntd.0002185 (PMC3642089; doi:10.1371/journal.pntd.0002185)
Supplement: File S4 — Other GO comparisons (Continued from Figure 5 ). A–C. Pair-wise comparisons of GO terms associated with Molecular Function among NILX, LPSX and LEISHX are shown. (PDF) [file pntd.0002185.s004.pdf]

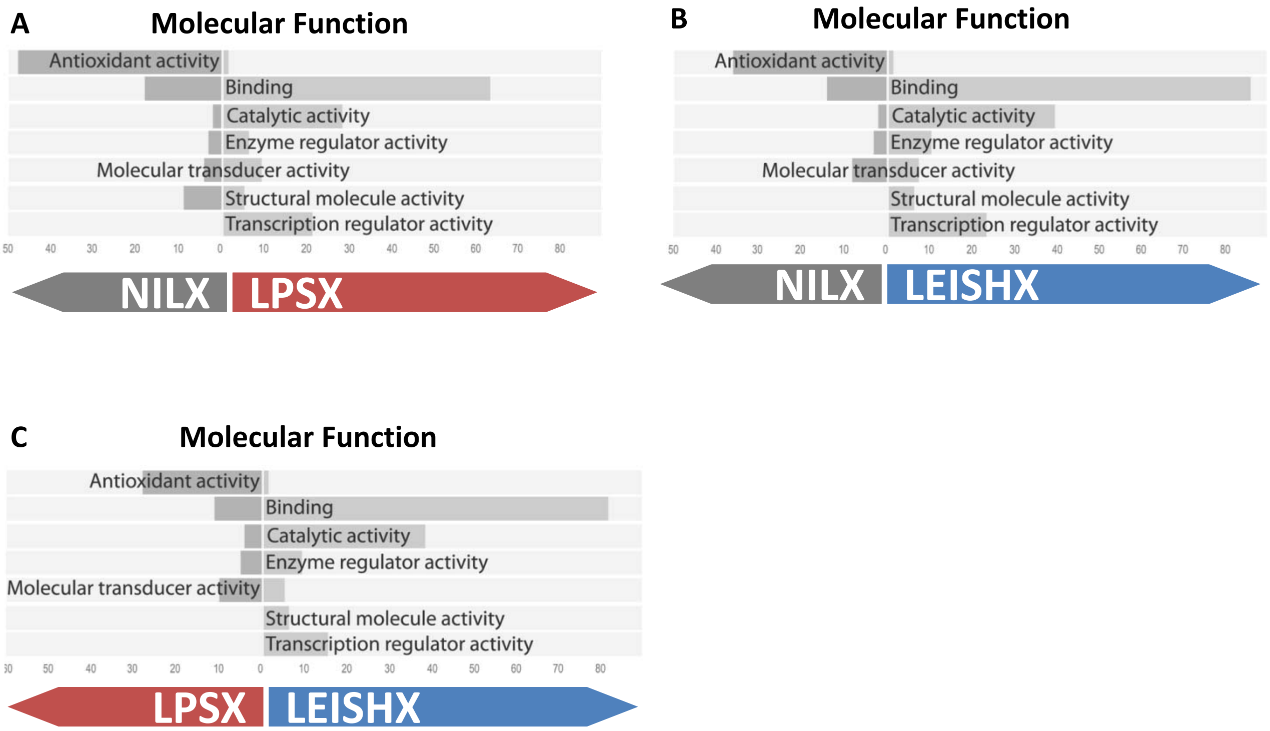


**Supplemental File S4. Other GO comparisons (Continued from Figure 5).** A-C. Pair-wise comparisons of GO terms associated with Molecular Function among NILX, LPSX and LEISHX are shown.
