# Supplementary material for: Immunomodulatory Impact of Leishmania-Induced Macrophage Exosomes: A Comparative Proteomic and Functional Analysis
Source: PLoS Negl Trop Dis. 2013 May 2;7(5):e2185. doi: 10.1371/journal.pntd.0002185 (PMC3642089; doi:10.1371/journal.pntd.0002185)
Supplement: File S5 — High resolution PPI networks of NILX, LPSX and LEISHX exosomal proteins. (PDF) [file pntd.0002185.s005.pdf]

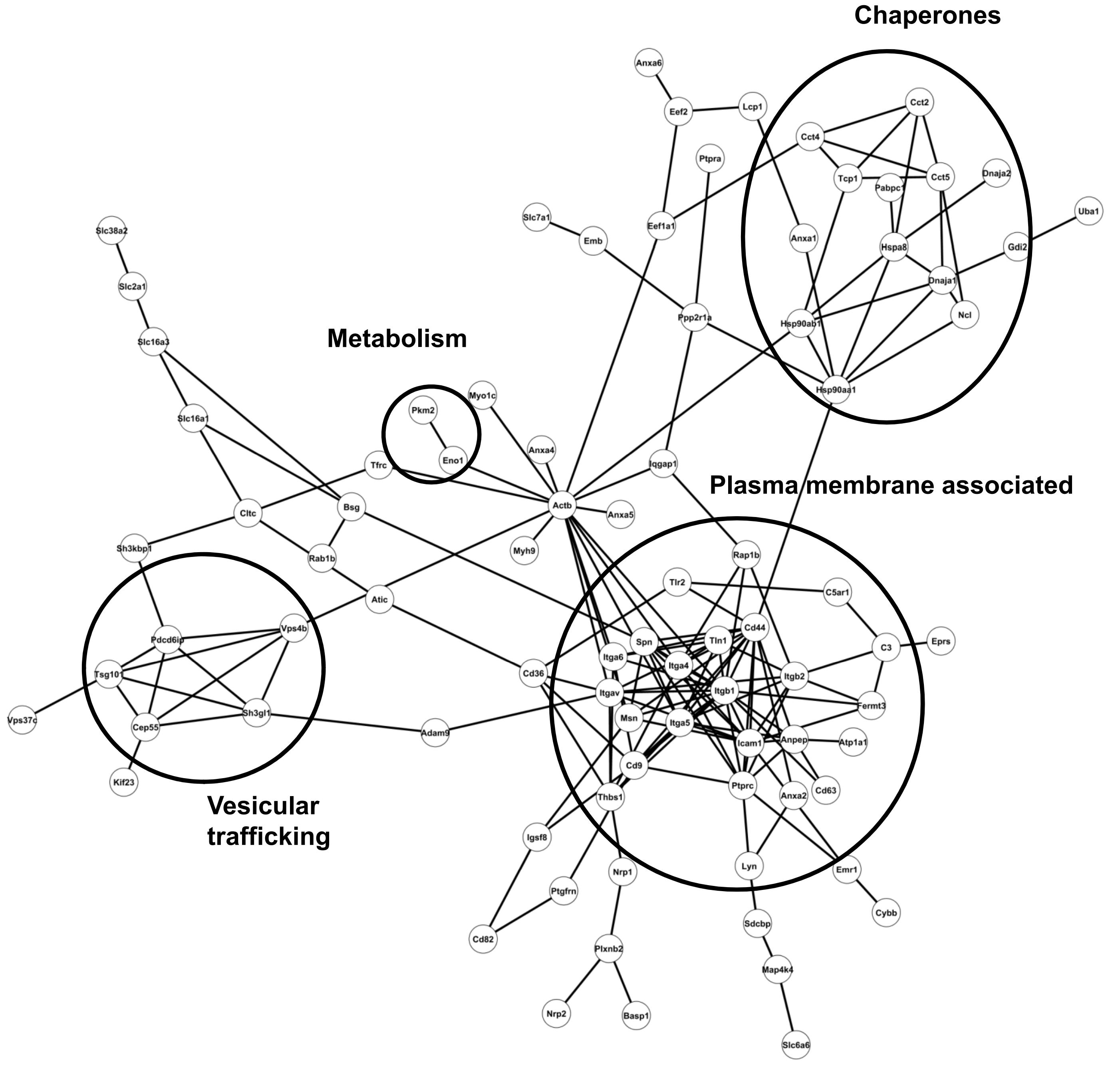

Supplemental File S5.
NILX PPI network and the interaction groups of proteins with related functions.

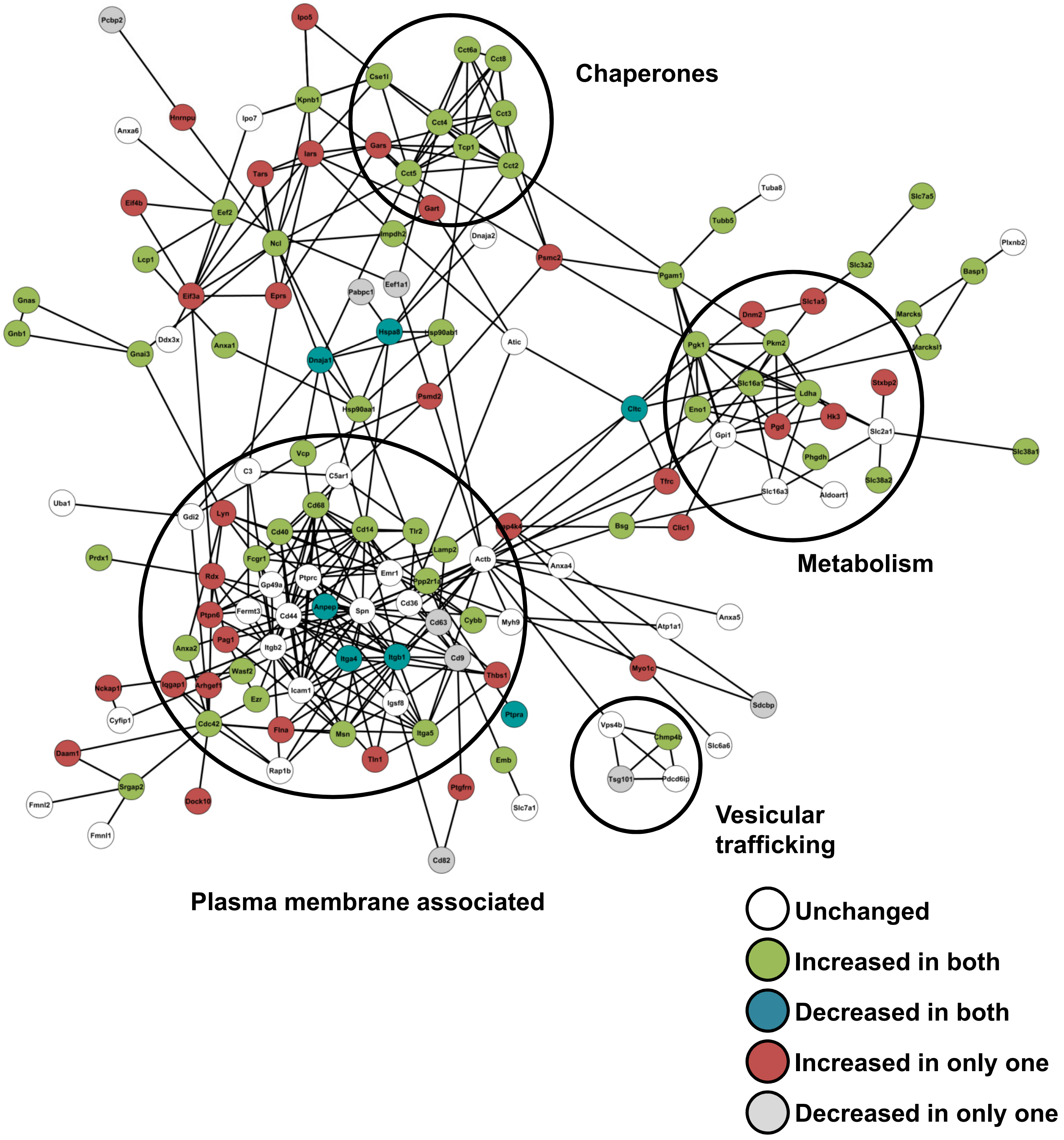

Supplemental File S5. Continued.
LEISHX PPI network and the interaction groups of proteins with related functions.

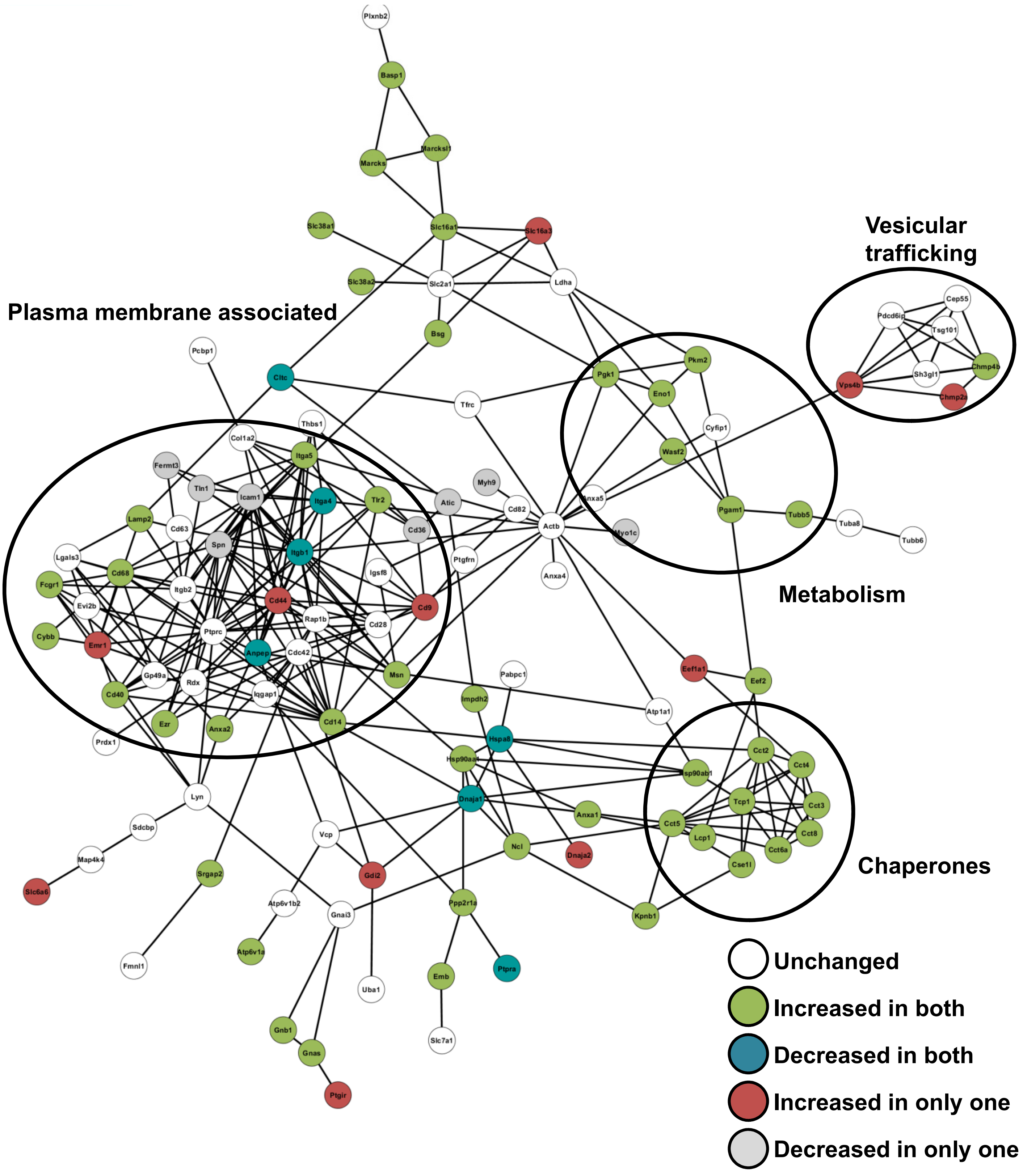

Supplemental File S5 Continued.
LPSX PPI network and the interaction groups of proteins with related functions.
